# The Association Between Gamma‐Glutamyl Transferase and Metabolic Syndrome and Its Components Among Adolescents Applying International Diabetes Federation (IDF) and Cook's Criteria

**DOI:** 10.1002/edm2.70074

**Published:** 2025-08-17

**Authors:** Farzad Esmaeili, Siavash Safiee, Mitra Hasheminia, Fereidoun Azizi, Maryam Tohidi, Farzad Hadaegh

**Affiliations:** ^1^ Prevention of Metabolic Disorders Research Center Research Institute for Metabolic and Obesity Disorders, Research Institute for Endocrine Sciences, Shahid Beheshti University of Medical Sciences Tehran Iran; ^2^ Faculty of Medicine Tehran Medical Branch, Islamic Azad University Tehran Iran; ^3^ Endocrine Research Center Research Institute for Endocrine Disorders, Research Institute for Endocrine Sciences, Shahid Beheshti University of Medical Sciences Tehran Iran

**Keywords:** adolescents, central obesity, gamma‐glutamyl transferase, metabolic syndrome

## Abstract

**Introduction:**

The prevalence of metabolic syndrome (MetS), a cluster of metabolic abnormalities, is rising globally, particularly in the Middle East and North Africa. Gamma‐glutamyl transferase (GGT) is gaining attention as a biomarker for liver function and its association with MetS and its components.

**Methods:**

This cross‐sectional study is part of the Tehran Lipid and Glucose Study (TLGS). We included 696 adolescents (347 males) aged 10–19 from the seventh examination survey (2018–2021). MetS was defined using both the International Diabetes Federation (IDF) and Cook's criteria. Serum GGT was measured, and its association as a continuous and categorical variable was assessed with MetS and its components using logistic regression, adjusting for a large set of covariates.

**Results:**

MetS prevalence was 15.66% and 9.19% according to Cook's and IDF criteria, respectively. Higher GGT levels were significantly associated with increased MetS risk by both definitions (odds ratio [95% confidence interval] = 1.28 [1.12–1.46] and 1.30 [1.14–1.49] per 5 U/L increase, respectively) after adjusting for age, sex, smoking and family history of type 2 diabetes mellitus. This association was attenuated upon adjusting for ALT levels. GGT levels were robustly associated with high waist circumference, with odds ratios of 1.98 [1.59–2.46] and 1.71 [1.38–2.11] per 5 U/L increase, respectively, even after adjusting for alanine aminotransferase (ALT). Associations with high blood pressure (21% and 17% increased risk by IDF and Cook's criteria) and triglycerides (13% and 16% increased risk by IDF and Cook's criteria) were significant but attenuated after ALT adjustment. No significant associations were found between GGT levels and high fasting plasma glucose or low high‐density lipoprotein cholesterol.

**Conclusions:**

Elevated serum GGT is strongly associated with a higher risk of MetS and its components, particularly central obesity, in adolescents. These findings suggest that GGT is a valuable biomarker for early MetS detection.

## Graphical abstract

Elevated serum gamma‐glutamyl transferase (GGT) levels were significantly associated with higher odds of metabolic syndrome and central obesity in adolescents. These findings highlight GGT as a potential early biomarker for cardiometabolic risk assessment in youth.

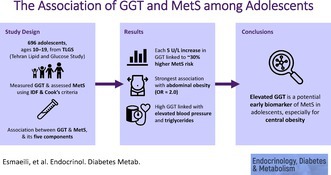

## 1 Introduction

Metabolic syndrome (MetS), a cluster of metabolic abnormalities including central obesity, high fasting blood glucose, dyslipidemia, and high blood pressure (BP), poses a major risk for cardiovascular diseases (CVD), type 2 diabetes mellitus (T2DM), and premature death, thus making it an emergent health issue globally [1, 2]. Latest reports indicate that among children and adolescents globally, 2.8% and 4.8%, respectively, were living with MetS in 2020, and an overall 390 million were overweight in 2022 [3, 4]. Notably, the prevalence of MetS varies significantly across regions, with Iran having one of the highest rates. Specifically, among adolescents, Iran's MetS prevalence stands at 9.0% (95% confidence interval [CI] 8.4–9.7), surpassing many other countries [3].

Dysregulated action of insulin in the liver, skeletal muscle, and adipose tissue, as the underlying cause of MetS, was linked with CVD, T2DM, as well as metabolic dysfunction‐associated steatotic liver disease (MASLD) [4, 5]; the newly redefined term for non‐alcoholic fatty liver disease which was introduced to be used interchangeably [6, 7]. Hepatic steatosis and subsequent MASLD are characterised by a high accumulation of lipids in hepatocytes independent of alcohol consumption and are associated with increased secretion of liver‐derived lipids, alterations in ketone production, and, finally, macrophage‐driven inflammation. Therefore, this could lead to lipid accumulation in skeletal muscle and adipose tissue. These pathological changes not only contribute to insulin resistance but also exacerbate atherogenic modifications, thereby increasing the risk of CVD [4].

The gold standard to diagnose MASLD is liver biopsy; however, assessing elevated serum transaminases could be a more affordable and non‐invasive path to evaluate liver function [8]. In this context, Gamma‐glutamyl transferase (GGT), an enzyme involved in glutathione and cysteine metabolism, has gained attention for its role in liver function assessment. GGT is physiologically abundant in the liver, kidney, pancreas, colon, and prostate, with serum levels rising in response to oxidative stress related to inflammation and dysregulation in lipid and glucose homeostasis [9]. GGT has both antioxidant and prooxidant functions, reflecting its complex role in oxidative stress [9, 10]. Historically, GGT has been used concurrently with alkaline phosphatase (ALP) as a beneficial biomarker for diagnosing liver cholestasis and assessing liver damage in alcoholic liver disease [9]. Supporting the potential role of inflammatory and metabolic mediators in MetS among youth, Galectin‐3, another biomarker, has been linked with insulin resistance and cardiovascular dysfunction in the early stages of T2DM pathophysiology [11]. Highlighting the importance of timely detection, the multisystem burden of early‐onset metabolic dysfunction was echoed by a recent meta‐analysis reporting a higher prevalence of comorbidities (including neurological, namely, restless leg syndrome) in diabetes [12]. In parallel, evidence suggests that greater therapeutic intensity in T2DM, such as combination regimens, may reflect a more advanced metabolic disease [13], reinforcing the importance of early identification strategies using accessible biomarkers like GGT. In recent works, the significant association of GGT with T2DM, CVD, and MetS was established in adults [14, 15, 16]. The overall relationship between GGT and MetS among adolescents has been investigated by studies, most of which were conducted in Europe [17, 18, 19, 20, 21, 22] and Asia [23, 24, 25, 26, 27]; however, among them, only a few have also examined the association of GGT with individual MetS components [20, 23, 25, 26].

Given the lack of a universally agreed‐upon definition for MetS in adolescents, our study employs the two most widely recognised criteria outlined by Cook et al. [28] and the International Diabetes Federation (IDF) [29]. Our objective is to explore the relationship between serum GGT levels and MetS and its components in adolescents in the Middle East and North Africa (MENA) region, applying both continuous and categorical approaches, the latter aimed at clinical purposes.

## 2 Materials and Methods

### 2.1 Study Design and Setting

The present study is a part of the Tehran Lipid and Glucose Study (TLGS), a longitudinal cohort designed to identify principal risk factors for non‐communicable diseases within a wide‐ranging population‐based sample in Tehran, Iran. From 1999 to 2001, the study enrolled 15,005 participants, aged three and up, from 13 districts of Tehran. The methodology and procedures of this study have been detailed in a prior publication [30]. After baseline recruitment, participants were followed by subsequent examinations at roughly 3‐year intervals. Individuals aged 10–19 years participated in the seventh examination survey (2018–2021) of TLGS and were included in this cross‐sectional study (*n* = 734). After excluding those with missing data on individual components of MetS and confounders (*n* = 38, considering overlap features between numbers), 696 adolescents (347 male) remained for data analysis.

The ongoing longitudinal prospective cohort study received ethical approval from the Research Institute for Endocrine Sciences (RIES) of Shahid Beheshti University of Medical Sciences (SBUMS) under the approval code IR.SBMU.ENDOCRINE.REC.1403.43011236. Following the Declaration of Helsinki principles, the study ensures a thorough briefing about the protocol for all participants. Written informed consent was obtained from all adult participants and parents or legal guardians of minors under 18.

### 2.2 Clinical and Laboratory Measurements

Initial data collection included socio‐demographic, lifestyle, and medical history information obtained through face‐to‐face interviews using a standardised questionnaire. Anthropometric measurements, including weight, height, and waist circumference (WC), were performed while minimally clothed and without shoes. WC was measured at the level of the umbilicus. BP, comprising systolic and diastolic BP (SBP and DBP, respectively), was measured after the subject had been seated for 15 min. Measurements were taken twice at 5‐min intervals from the right brachial artery at heart level, calibrated by the Iranian Institute of Standards and Industrial Research using a standard mercury sphygmomanometer and the correct cuff size. The average of these two readings was recorded as the individual's blood pressure [30].

Blood samples for biochemical analyses were collected in the morning after an overnight fast between 7 and 9 Am. After centrifugation, some serum samples were stored at −80°C for later analyses. Measurement of fasting plasma glucose (FPG), lipids including triglycerides (TG), high‐density lipoprotein cholesterol (HDL‐C), aspartate aminotransferase (AST), and alanine aminotransferase (ALT) was performed on the same day as sampling. Glucose was assayed by an enzymatic colorimetric method using glucose oxidase. TG levels were determined using an enzymatic colorimetric method with glycerol phosphate oxidase. HDL‐C was quantified using an enzymatic calorimetric method involving cholesterol esterase and cholesterol oxidase. AST and ALT were measured using enzymatic photometry. All these biochemical parameters were assayed using commercial kits (Pars Azmoon, Iran) and a Selectra 2 chemistry auto‐analyser (Vital Scientific, the Netherlands). Serum GGT levels were determined in the stored sample by a colorimetric method using commercial kits from Delta Darman Part Inc., Iran, on a Pictus 700 auto‐analyser (Diatron Inc., Budapest, Hungary). The intra‐assay and inter‐assay coefficients of variation (CVs) of liver enzymes were 3.4% and 4.8% for AST, 2.6% and 5.4% for ALT, and 2.6% and 3.6% for GGT, respectively. Both the intra‐assay and inter‐assay CVs for glucose, TG, and HDL‐C were less than 3.8%. To monitor the quality of assays, we used commercial serum controls in two different concentrations in the following manner: those from Pars Azmoon Inc., Iran, for glucose and lipid assays, and those from Delta Darman Part Inc., Iran, for measurement of liver enzymes.

### 2.3 Definitions

A current smoker was described as a person smoking cigarettes or other related implements daily or occasionally. Ever‐smokers were described as those who had quit smoking for at least 1 year before assessment or individuals who had never smoked. Body mass index (BMI) was computed as weight in kg divided by height in square meters (kg/m^2^). A family history of T2DM was described as having at least one first‐degree relative (parent and sibling) diagnosed with T2DM.

In this study, MetS was defined according to two widely recognised criteria specifically adapted for adolescents. The IDF consensus of 2007 for adolescents aged 10–16 years diagnosed MetS when WC was established to be high according to a national study among Iranian adolescents [31], accompanied by at least two of the following risk factors: elevated BP of 130/85 mmHg or higher, HDL‐C lower than 40 mg/dL, elevated TG of 150 mg/dL or higher, and FPG equal to or higher than 100 mg/dL or known T2DM [29]. For individuals aged 16–19, the IDF Consensus 2005 was used accordingly [32]. Also, the updated version from the National Cholesterol Education Program Adult Treatment Panel III (NCEP‐ATP III), Cook et al. criteria, was used [28]. According to it, MetS was defined on the basis of the presence of three or more of the following: WC at or above the 90th percentile for age and gender [33], TG of 110 mg/dL or higher, HDL‐C of 40 mg/dL or lower, BP at or above the 90th percentile for age, gender and height [28] and FPG of 100 mg/dL or higher according to the most recent guidelines of American Diabetes Association [34].

2.4

Baseline characteristics for the entire population (*n* = 696) and for those with and without MetS were displayed as mean ± standard deviation (SD) for continuous variables with a normal distribution, median (interquartile range [IQR]) for continuous variables with a high degree of skewness, and frequency (%) for categorical variables. Differences in baseline characteristics between the groups were analysed using the *t*‐test, the Mann–Whitney test, and the chi‐square test as suitable. Logistic regression was utilised to investigate the association between GGT levels and MetS, along with its components. Odds ratios (ORs) and 95% CIs were computed to quantify the strength and precision of the associations. GGT was treated as both a continuous variable (analysed for its effects per 5 U/L increase) and a categorical variable (i.e., the individuals with < 75th percentile of serum GGT levels were considered the reference, as used by previous literature [23, 25]).

The analytical approach was structured through a series of models designed to adjust for various confounders sequentially. To assess the association of GGT with MetS, the models were as follows: Model I, adjusted for age and sex; Model II expanded these adjustments to include smoking status and family history of T2DM. Moreover, for each MetS component, we ran Model III by adding four other components. Finally, we also examined the association between GGT and MetS, as well as its individual components, with further adjustment for ALT in Model IV (i.e., Model II plus ALT for MetS, and Model III plus ALT, for MetS components).

Statistical significance was established at a *p*‐value less than 0.05 using a two‐tailed test. All statistical procedures were performed using SPSS software (version 26.0) and STATA software (version 17.0), ensuring rigorous data evaluation according to established clinical research standards.

## 3 Results

**FIGURE 1:**
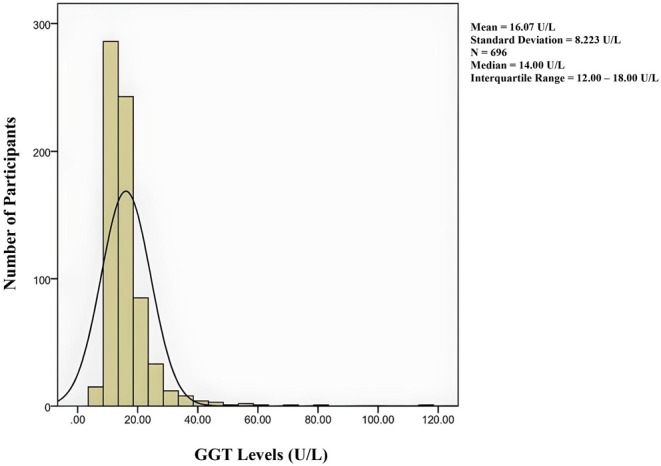
The distribution of GGT levels in the study population (*n* = 696).

Our study included 696 adolescents (347 male) aged between 10 and 19 years, with a mean ± SD age of 15.08 ± 2.50. As illustrated in Table 1, the population characteristics show that out of 696 subjects, 64 (9.19%) and 109 (15.66%) were diagnosed with MetS according to IDF and Cook's definitions, respectively. Individuals with MetS had higher BMI, WC, SBP, DBP, TG, and lower HDL‐C (all *p*‐values < 0.001). Nonetheless, significantly higher FPG was only observed in individuals with MetS, defined by Cook's criteria (*p*‐value = 0.020). Considering liver enzymes, GGT, AST, and ALT levels were shown to be significantly higher among individuals with MetS, regardless of the definition (all *p*‐values < 0.001). The smoking status and family history of T2DM were not significantly different between the two groups by either criterion. Also, the distribution of GGT levels in the study population has been demonstrated in Figure 1.

**TABLE 1:** Characteristics of the study participants by MetS status according to IDF and Cook's definitions, TLGS (2018–2021).

Variables	Whole population (*n* = 696)		MetS defined according to IDF criteria			MetS defined according to Cook's criteria		
			MetS (+) (*n* = 64)	MetS (−) (*n* = 632)	*p*	MetS (+) (*n* = 109)	MetS (−) (*n* = 587)	*p*
**Demographics**								
Age, year	15.08 ± 2.5		15.45 ± 2.33	15.04 ± 2.51	0.211	14.91 ± 2.44	15.11 ± 2.51	0.434
Sex (male%)	347 (49.9)		36 (56.2)	31 (5.0)	0.297	59 (54.1)	288 (49.1)	0.359
BMI (kg/m^2^)	23.11 ± 5.16		28.53 ± 3.77	22.56 ± 4.97	< 0.001	27.59 ± 4.46	22.28 ± 4.85	< 0.001
WC (cm)	80.69 ± 13.04		95.69 ± 8.94	79.17 ± 12.42	< 0.001	92.86 ± 11.37	78.42 ± 12.05	< 0.001
SBP (mmHg)	103.64 ± 12.1		113.36 ± 13.1	102.65 ± 11.56	< 0.001	110.6 ± 14.02	102.34 ± 11.26	< 0.001
DBP (mmHg)	69.16 ± 9.37		75.06 ± 9.11	68.56 ± 9.19	< 0.001	74.24 ± 9.66	68.22 ± 9.01	< 0.001
FPG (mg/dL)	92.01 ± 17.64		95.6 ± 8.4	91.6 ± 18.3	0.085	98.8 ± 35.9	90.8 ± 11.0	0.020
TG (mg/dL)^a^	87.0 (64.0–121.0)		166.0 (144.0–199.0)	84.0 (62.0–111.0)	< 0.001	156.0 (121.5–188.0)	81.0 (60.0–103.0)	< 0.001
HDL‐C (mg/dL)	47.83 ± 9.5		38.8 ± 6.4	48.7 ± 9.30	< 0.001	40.2 ± 8.3	49.3 ± 9.0	< 0.001
GGT (U/L)^a^	14.0 (12.0–18.0)		19.0 (15.0–24.0)	14.0 (12.0–17.0)	< 0.001	18.0 (13.0–21.0)	14.0 (12.0–17.0)	< 0.001
AST (U/L)	24.22 ± 8.41		29.2 ± 14.6	23.7 ± 7.4	0.004	27.9 ± 13.3	23.5 ± 6.9	0.001
ALT (U/L)	18.35 ± 15.04		29.2 ± 23.7	17.2 ± 13.5	< 0.001	27.2 ± 21.8	16.7 ± 12.8	< 0.001
Smoking (yes%)	158 (25.9)		16 (29)	142 (22.5)	0.297	29 (26.6)	129 (22)	0.319
FH‐T2DM (yes%)	99 (14.2)		12 (18.8)	87 (13.8)	0.264	18 (16.5)	81 (18.8)	0.457
**MetS components**	**IDF**	**Cook's**						
High WC (yes%)	330 (47.4)	326 (46.8)	64 (100)	266 (42.1)	< 0.001	100 (91.7)	226 (38.5)	< 0.001
High BP (yes%)	38 (5.5)	63 (9.1)	17 (26.6)	21 (3.3)	< 0.001	38 (34.9)	25 (4.3)	< 0.001
High FPG (yes%)	92 (13.2)	90 (12.9)	23 (35.9)	69 (10.9)	< 0.001	38 (34.9)	5.2 (8.9)	< 0.001
High TG (yes%)	102 (14.7)	207 (29.7)	48 (75)	54 (8.5)	< 0.001	99 (90.8)	108 (18.4)	< 0.001
Low HDL‐C (yes%)	184 (26.4)	153 (22.0)	49 (76.6)	135 (21.4)	< 0.001	73 (67)	80 (13.6)	< 0.001

*Note:* Data are shown as mean ± SD, or *n* (percentage).

Abbreviations: ALT, alanine aminotransferase; AST, aspartate aminotransferase; BMI, body mass index; DBP, diastolic blood pressure; FH‐T2DM, family history of type 2 diabetes mellitus; FPG, fasting plasma glucose; GGT, gamma‐glutamyl transferase; HDL‐C, high‐density lipoprotein cholesterol; IDF, International Diabetes Federation; MetS, metabolic syndrome; SBP, systolic blood pressure; SD, standard deviation; TG, triglycerides; TLGS, Tehran Lipid and Glucose Study; WC, waist circumference.

^a^ Data with skewed distribution are shown as median (interquartile range).

Tables 2 and 3 show the associations of serum GGT, analysed as a continuous variable, with MetS and its components by IDF and Cook's criteria, respectively. Increased levels of GGT were significantly associated with MetS by both definitions, harbouring about 30% higher risks after adjustment for age, sex, smoking, and T2DM family history (OR [95% CI] = 1.28 [1.12–1.46] and 1.30 [1.14–1.49] for a 5 U/L increase in GGT according to IDF and Cook's criteria, respectively in model II, with both *p*‐values < 0.001); these relationships significantly reduced after further adjustment for ALT (Model III). Importantly, in this fully adjusted model, ALT levels had a significant association with prevalent MetS (1.02 [1.01–1.04] and 1.04 [1.02–1.06], according to IDF and Cook's criteria, respectively). Regarding the MetS components, increased GGT significantly doubled the risk of high WC even after adjusting for other MetS components (Model III), using both definitions. Although the risks were slightly attenuated when adjusted for ALT levels (Model IV), the associations remained significant (1.98 [1.59–2.46] and 1.71 [1.38–2.11] for a 5 U/L increase in GGT according to IDF and Cook's criteria, respectively; with both *p*‐values < 0.001). For high BP component, we also found that 5 U/L increase in GGT was associated with 21% (*p*‐value = 0.011) and 17% (*p*‐value = 0.011) increased risk in Model III given IDF and Cook's criteria, respectively; the comparable values for high TG component were 13% (*p*‐value = 0.051) and 16% (*p*‐value = 0.023), respectively (all *p*‐values were statistically significant, excluding the high TG for IDF criteria that tended to be significant). However, the associations were markedly attenuated when further adjustment for ALT levels was applied. High FPG and low HDL‐C components did not show a significant association in any of the models.

**TABLE 2:** OR (95% CI) for the associations between GGT and MetS and its components according to IDF definition, TLGS (2018–2021).

	High WC (*N* = 330)	High BP (*N* = 38)	High FPG (*N* = 92)	High TG (*N* = 102)	Low HDL‐C (*N* = 184)	MetS (*N* = 64)
GGT (per 5 U/L increase)						
Model I	2.35 (1.92–2.88)	1.27 (1.10–1.47)	1.06 (0.93–1.21)	1.22 (1.08–1.38)	1.02 (0.92–1.13)	1.28 (1.12–1.47)
*p*‐value	< 0.001	< 0.001	0.324	< 0.001	0.624	< 0.001
Model II	2.35 (1.91–2.87)	1.27 (1.09–1.47)	1.05 (0.92–1.2)	1.21 (1.08–1.37)	1.02 (0.92–1.13)	1.28 (1.12–1.46)
*p*‐value	< 0.001	0.001	0.421	0.001	0.659	< 0.001
Model III	2.22 (1.81–2.73)	1.21 (1.04–1.41)	0.99 (0.84–1.16)	1.13 (1.00–1.28)	0.89 (0.77–1.02)	N/A
*p*‐value	< 0.001	0.011	0.930	0.051	0.090	
Model IV^a^	1.98 (1.59–2.46)	1.14 (0.95–1.36)	1.01 (0.84–1.22)	1.03 (0.89–1.20)	0.9 (0.77–1.05)	1.11 (0.96–1.30)
*p*‐value	< 0.001	0.143	0.882	0.622	0.192	0.139

*Note:* Model I: adjusted for age and sex. Model II: model I + smoking status and family history of type 2 diabetes mellitus. Model III: model II + other MetS components. Model IV: Model III + ALT.

Abbreviations: ALT, alanine aminotransferase; BP, blood pressure; CI, confidence interval; FPG, fasting plasma glucose; GGT, gamma‐glutamyl transferase; HDL‐C, high‐density lipoprotein cholesterol; IDF, international diabetes federation; MetS, metabolic syndrome; N/A, not applicable; OR, odds ratio; TG, triglycerides; TLGS, Tehran lipid and glucose study; WC, waist circumference.

^a^ Model IV for incident MetS: Model II + ALT.

**TABLE 3:** OR (95% CI) for the associations between GGT and MetS and its components according to Cook's definition, TLGS (2018–2021).

	High WC (*N* = 326)	High BP (*N* = 63)	High FPG (*N* = 90)	High TG (*N* = 207)	Low HDL‐C (*N* = 153)	MetS (*N* = 109)
GGT (per 5 U/L increase)						
Model I	2.23 (1.83–2.71)	1.23 (1.08–1.40)	1.06 (0.92–1.21)	1.32 (1.16–1.49)	1.05 (0.94–1.16)	1.31 (1.15–1.49)
*p*‐value	< 0.001	0.001	0.390	< 0.001	0.350	< 0.001
Model II	2.23 (1.83–2.71)	1.24 (1.08–1.42)	1.05 (0.91–1.20)	1.33 (1.17–1.51)	1.04 (0.94–1.15)	1.30 (1.14–1.49)
*p*‐value	< 0.001	0.001	0.491	< 0.001	0.409	< 0.001
Model III	1.99 (1.63–2.43)	1.17 (1.02–1.34)	0.97 (0.82–1.16)	1.16 (1.02–1.32)	0.93 (0.81–1.06)	N/A
*p*‐value	< 0.001	0.011	0.778	0.023	0.301	
Model IV^a^	1.71 (1.38–2.11)	1.09 (0.93–1.29)	1.00 (0.81–1.22)	1.09 (0.94–1.26)	0.93 (0.79–1.08)	1.08 (0.93–1.25)
*p*‐value	< 0.001	0.238	0.972	0.622	0.363	0.289

*Note:* Model I: adjusted for age and sex. Model II: model I + smoking status and family history of type 2 diabetes mellitus. Model III: model II + other MetS components. Model IV: Model III + ALT.

Abbreviations: ALT, alanine aminotransferase; BP, blood pressure; CI, confidence interval; FPG, fasting plasma glucose; GGT, gamma‐glutamyl transferase; HDL‐C, high‐density lipoprotein cholesterol; MetS, metabolic syndrome; N/A, not applicable; OR, odds ratio; TG, triglycerides; TLGS, Tehran lipid and glucose study; WC, waist circumference.

^a^ Model IV for incident Mets: Model II + ALT.

Tables 4 and 5 demonstrated the associations between elevated GGT with MetS and its components by IDF and Cook's criteria, respectively. Generally, the results of the categorical analysis of GGT were aligned with those of the continuous analysis. Accordingly, high GGT was significantly associated with prevalent MetS, using both IDF (OR [95% CI]: 4.44 [2.40–8.23]) and Cook's criteria (3.50 [2.11–5.78]; with both *p*‐values < 0.001), even after ALT Adjustment. Moreover, high GGT was associated with both abdominal obesity and high TG components of MetS in the fully adjusted model.

**TABLE 4:** OR (95% CI) for the associations between high GGT^a^ and MetS and its components according to IDF definition, TLGS (2018–2021).

	High WC	High BP	High FPG	High TG	Low HDL‐C	MetS
The reference group: GGT < 18 U/L						
Model I						
GGT ≥ 18 U/L	5.29 (3.53–7.93)	2.77 (1.36–5.61)	1.61 (0.98–2.66)	3.87 (2.42–6.18)	1.43 (0.94–2.16)	5.74 (3.23–10.21)
*p*‐value	< 0.001	0.026	0.443	< 0.001	0.286	< 0.001
Model II						
GGT ≥ 18 U/L	5.27 (3.51–7.90)	2.74 (1.35–5.57)	1.56 (0.94–2.59)	3.83 (2.39–6.12)	1.42 (0.93–2.15)	5.68 (3.19–10.11)
*p*‐value	< 0.001	0.030	0.366	< 0.001	0.172	< 0.001
Model III						
GGT ≥ 18 U/L	4.6 (3.2–7.00)	1.95 (0.9–4.22)	1.28 (0.74–2.22)	2.79 (1.65–4.73)	0.8 (0.5–1.29)	N/A
*p*‐value	< 0.001	0.184	0.798	0.002	0.741	
Model IV^b^						
GGT ≥ 18 U/L	3.20 (2.05–5.00)	1.59 (0.70–3.60)	1.36 (0.76–2.41)	2.43 (1.40–4.23)	0.86 (0.52–1.42)	4.44 (2.40–8.23)
*p*‐value	0.001	0.313	0.734	0.017	0.852	< 0.001

*Note:* Model I: adjusted for age and sex. Model II: model I + smoking status and family history of type 2 diabetes mellitus. Model III: model II + other MetS components. Model IV: Model III + ALT.

Abbreviations: ALT, alanine aminotransferase; BP, blood pressure; CI, confidence interval; FPG, fasting plasma glucose; GGT, gamma‐glutamyl transferase; HDL‐C, high‐density lipoprotein cholesterol; IDF, international diabetes federation; MetS, metabolic syndrome; N/A, not applicable; OR, odds ratio; TG, triglycerides; TLGS, Tehran lipid and glucose study; WC, waist circumference.

^a^ ≥ 75th percentile of GGT levels.

^b^ Model IV for incident Mets: Model II + ALT.

**TABLE 5:** OR (95% CI) for the associations between high GGT^a^ and MetS and its components according to Cook's definition, TLGS (2018–2021).

	High WC	High BP	High FPG	High TG	Low HDL‐C	MetS
The reference group: GGT < 18 U/L						
Model I						
GGT ≥ 18 U/L	5.00 (3.35–7.44)	2.28 (1.30–3.99)	1.56 (0.94–2.59)	4.08 (2.74–6.07)	1.65 (1.084–2.51)	4.89 (3.09–7.76)
*p*‐value	< 0.001	0.014	0.393	< 0.001	0.075	< 0.001
Model II						
GGT ≥ 18 U/L	5.00 (3.35–7.48)	2.28 (1.30–4.00)	1.52 (0.91–2.52)	4.12 (2.76–6.13)	1.63 (1.07–2.48)	4.86 (3.06–7.71)
*p*‐value	< 0.001	0.015	0.456	< 0.001	0.082	< 0.001
Model III						
GGT ≥ 18 U/L	3.83 (2.52–5.83)	1.48 (0.80–2.73)	1.16 (0.66–2.02)	2.81 (1.80–4.37)	0.97 (0.60–1.55)	N/A
*p*‐value	< 0.001	0.216	0.913	< 0.001	0.885	
Model IV^b^						
GGT ≥ 18 U/L	2.54 (1.62–3.97)	1.21 (0.63–2.32)	1.23 (0.69–2.21)	2.59 (1.63–4.13)	1.01 (0.61–1.69)	3.50 (2.11–5.78)
*p*‐value	< 0.001	0.322	0.876	0.001	0.882	< 0.001

*Note:* Model I: adjusted for age and sex. Model II: model I + smoking status and family history of type 2 diabetes mellitus. Model III: model II + other MetS components. Model IV: Model III + ALT.

Abbreviations: ALT, alanine aminotransferase; BP, blood pressure; CI, confidence interval; FPG, fasting plasma glucose; GGT, gamma‐glutamyl transferase; HDL‐C, high‐density lipoprotein cholesterol; MetS, metabolic syndrome; N/A, not applicable; OR, odds ratio; TG, triglycerides; TLGS, Tehran lipid and glucose study; WC, waist circumference.

^a^ ≥ 75th percentile of GGT levels.

^b^ Model IV for incident Mets: Model II + ALT.

## 4 Discussion

Our findings revealed that a 5 U/L increase in GGT was significantly correlated with a 30% higher risk for prevalent MetS, as defined by both IDF and Cook's criteria, even after adjusting for age, sex, smoking status, and family history of T2DM. This association significantly attenuated after adjusting for ALT levels. Regarding the MetS components, we found a robust association between GGT and central adiposity in the fully adjusted model, using both MetS definitions. Importantly, applying a categorical approach, we found that GGT ≥ 18 U/L (≥ 75th percentile) was significantly associated with prevalent MetS, high TG, and abdominal obesity in the multivariate analyses, adjusting for ALT, regardless of MetS definition criteria.

In the current study, we found that the prevalence of MetS among Tehranian adolescents in 2022 was 9.19% and 15.6%, according to IDF and Cook's definitions, respectively. In a review of studies published from 2014 to 2019, Reisinger et al. reported that the prevalence of MetS ranged between 0.3% and 26.4%, an issue that partly depended on the definition used [35]. As expected, the IDF definition generally provided the lowest prevalence (0.3%–9.5%) [29], whereas the classification of de Ferranti et al. yielded the highest (4.0%–26.4%) [36]. Recently, according to a systematic review and modelling analysis, using the presence of three or more of five components to define the MetS, Iran, the United Arab Emirates, and Spain have the highest prevalence of MetS in adolescents, values of which reached more than 9.0% [3].

The association between GGT and MetS has been investigated mostly in European and Asian populations, and to our knowledge, no studies on the topic have been conducted in the MENA region. The studies on adolescents found the potential association of GGT with obesity/MetS and the MetS components, with inconsistent results. Importantly, comparing our findings with other studies in this field is not simple, considering factors such as the age of participants, ethnicity, level of adjustment for confounders, and the recruitment source of study participants (clinic vs. general population) or the study participants being limited to a specified subgroup, such as the obese ones.

As for the research conducted on European populations, in a clinic‐based study among 132 obese Spanish adolescents, those with MetS (IDF criteria) did not have higher liver enzymes, including GGT, compared with non‐MetS individuals. However, only ALT levels were significantly associated with a number of MetS components [20]. In another clinic‐based study on 126 Serbian overweight/obese participants aged 15–26, the levels of GGT and ALT were higher among those with MetS (Cook's criteria) compared with non‐MetS individuals; however, the presence of MetS per se was not correlated with these enzymes as dependent variables in the regression model [19]. In a study on 100 Polish obese adolescents, it was shown that besides WC and total cholesterol levels, GGT was significantly differentiated among adolescents suffering from MASLD from obese ones without this condition. Moreover, in this study, the highest discriminatory power for differentiating MASLD from non‐MASLD obese adolescents was related to GGT [22]. In a recently published cross‐sectional study also conducted on 99 Polish adolescents, the authors found that fasting ALT was a better positive predictor for prevalent MetS (Cook's criteria) than GGT; the corresponding values for a 1 U/L increase in these liver enzymes were 25% and 9%, respectively [17]. Similarly, in our data analysis, the 16% increased risk of MetS for a 5 U/L increase in GGT reached null after further adjustments for ALT levels. However, ALT itself remained significant with prevalent MetS. In an adiposity follow‐up registry among children aged 8–18 years with overweight or obesity, a strong correlation was found between central adiposity, as measured by waist‐to‐height ratio and GGT levels; this association was strongest among prepubertal children [21]. In our multivariable analysis among Iranian adolescents, we found that a 5 U/L increment in GGT levels was associated with a doubled risk of central adiposity, on the basis of both MetS definitions, independent of a large set of covariates, including ALT.

In the context of research on Asian populations, in an analysis of MetS (IDF criteria) on a group of 2067 Hong Kong children and adolescents aged 6–20 years, the authors found that individuals in the upper quartile (> 20 and 16 IU/L for boys and girls, respectively) for GGT were significantly associated with abdominal obesity, high TG, low HDL‐C, and high BP [23]. Similar to our findings, the authors did not find a significant association between GGT and dysglycaemia [23]. Furthermore, in a study conducted on 7072 Taiwanese adolescents aged 10–15, the authors found that WC and TG levels were more strongly associated with GGT among the whole population. In the prospective phase, they also demonstrated that high GGT levels were associated with about a 2‐fold increased risk of MetS (IDF criteria) [26]. Moreover, in a study conducted in the framework of the Fifth Korean National Health and Nutrition Examination Survey (KNHANES) on 1618 Korean adolescents aged 10–18 years, high GGT had significant associations with MetS (IDF criteria) in the whole population (OR = 5.79 for male, and 6.20 for female), comparable to our findings. As for the Mets components, high GGT, as defined by the values ≥ 75th percentile, was also significantly associated with all components except for low HDL‐C in males and high BP in females [25]. We extended the above studies by showing that participants in the upper quartile for GGT (i.e., values ≥ 18 U/L) had significantly higher risks for prevalent MetS, high TG, and abdominal obesity, regardless of employed MetS criteria in the presence of a much more comprehensive set of confounders, including ALT levels.

As the underlying association between GGT and MetS, it is suggested that by transferring glutamyl groups from gamma‐glutamyl peptides to other peptides and catabolising glutathione, GGT fuels the availability of cysteine, glutathione resynthesis, and plays a significant detoxication role in cells afterward [37, 38, 39]. Thereby, GGT is elevated in subclinical inflammation as it was featured in oxidative stress and homeostasis of glutathione [37]. The state of oxidative stress of MetS is also a linkage between elevated GGT and pro‐inflammatory factors [40], including tumour necrotising factor‐α [40, 41], interleukin‐6 [42], and C‐reactive protein [43], although the role of causality of GGT in MetS is not yet fully investigated [44]. The interplay between liver enzymes and systemic inflammation in MetS gains is further supported by the fact that other biomarkers, like Galectin‐3, a macrophage‐derived biomarker implicated in insulin resistance and β‐cell apoptosis, are associated with multiple diabetic micro‐ and macro‐vascular complications, via oxidative stress pathways [11]. As a nod to the significance of early assessment of metabolic dysfunction, the broader complications of metabolic disturbance have been highlighted by the high prevalence of even neurological complications in diabetes [12]. Moreover, research suggesting increased mortality risks with intensified T2DM therapy reflects the clinical burden of advanced metabolic disease [13], further underscoring the value of early‐phase biomarkers like GGT in identifying at‐risk youth before pharmacologic escalation becomes necessary.

In terms of clinical implications, evidence from paediatric cohorts in different parts of the world [45, 46, 47], in line with our results, demonstrates that elevated GGT is associated with key MetS components such as central obesity and dyslipidaemia. These findings may carry practical relevance for early risk assessment in adolescent populations. Furthermore, GGT, as a predictor or even surrogate of MASLD [48, 49], could be useful in screening participants when looking through the lens of MetS risk stratification. Therefore, the potential role of GGT must be weighed against the cost and accessibility issues of its measurement for identifying high‐risk adolescents in regions with a high burden of MetS.

Our study has several strengths, enhancing the robustness of our findings. Firstly, using both IDF and Cook's criteria provided robust comparisons across definitions, and our detailed MetS component analysis offered a nuanced understanding of GGT's metabolic impact. Secondly, focusing on a population of adolescents from the MENA region fills a critical gap in the literature. However, our study also had certain limitations. First, the cross‐sectional design limits causal inferences, and single‐time‐point measurements of GGT may not capture intra‐individual variability. Second, our data analysis did not consider important residual confounders such as adolescents' dietary habits and physical activity.

In conclusion, our study among Iranian adolescents showed that high serum GGT, as a surrogate of MASLD, was strongly associated with prevalent MetS, abdominal obesity, and high TG, independent of ALT levels, using both IDF and Cook's criteria.

## Author Contributions

F.E. contributed to the formal analysis and methodology, as well as the writing and review of the original draft. S.S. contributed to the formal analysis, methodology, writing and review of the original draft. M.H. contributed to the data curation, formal analysis, writing and review of the original draft. F.A. contributed to the study supervision, project administration and validation. M.T. contributed to the study conceptualisation, data curation, supervision, project administration, funding acquisition, writing and review of the original draft. F.H. contributed to the study conceptualisation, methodology, supervision, writing and review of the original draft. All authors read, reviewed, and approved of the final manuscript.

## Conflicts of Interest

The authors declare no conflicts of interest.

## Data Availability

The datasets analysed during the current study are not publicly available because of privacy concerns and ethical restrictions, but they are available from the corresponding author upon reasonable request.
